# Bonding of Al6061 by Hot Compression Forming: A Computational and Experimental Study of Interface Conditions at Bonded Surfaces

**DOI:** 10.3390/ma14133598

**Published:** 2021-06-28

**Authors:** Brigit Mittelman, Michael Ben-Haroush, Ira Aloush, Linoy Mordechay, Elad Priel

**Affiliations:** 1 Department of Mechanical Engineering, Center for Thermo-Mechanics and Failure of Materials, Shamoon Collage of Engineering, Bee’r-Sheva 84100, Israel; sikora.ira92@gmail.com (I.A.); linoy1101@gmail.com (L.M.); 2 Department of Materials, Nuclear Research Center Negev (NRCN), Bee’r-Sheva 84190, Israel; mikib789@gmail.com

**Keywords:** bonding strength, compression, forming, Al6061, FE

## Abstract

In recent years, there has been a growing interest in composite components, which may be designed to provide enhanced mechanical and physical effective properties. One of the methods available to produce such components is joining by plastic deformation, which results in metallurgical bonding at the interface. However, the portions of the interface that are bonded and the inhomogeneity in the bonding strength achieved at the interface tend to be overlooked. In the present study, Al6061 beams were bonded, by hot compression (300–500 °C) to different degrees of reduction. The compression was followed by tensile debonding experiments and the revealed interface was microscopically characterized in order to determine the areas that were metallurgically bonded. The SEM characterization revealed that the actual bonded area is much smaller than the interface contact area. Thermo-mechanical finite element models of the compression stage were used to investigate the thermo-mechanical fields, which develop along the interface and influence the resulting bonding strength. The principal strain field patterns across the interface area were shown to be similar to the experimentally observed temperature-dependent bonding patterns. In addition, a quantitative criterion for bonding quality was implemented and shown to correlate with the experimental findings.

## 1. Introduction

Modern engineering components used in the aerospace, automotive and nuclear industries are designed to function under greater operating loads and harsher environmental conditions [1,2]. This is possible due to the incorporation of new and innovative composite materials, which provide enhanced mechanical and physical effective properties [3]. One class of composite components are multi-layered components, in which dissimilar or similar materials are joined to create one metallic component [4,5,6]. Joining processes can be classified into the following two groups: mechanical joining (such as by using fasteners, and clinching), and joining that includes metallurgical bonding (such as welding and adhesive bonding) [7]. The use of aluminum alloys for structural component manufacturing for those industries is increasing, due to their characteristic high strength-to-weight ratio, excellent formability and corrosion resistance. Among the widely used aluminum alloys is Al 6061, which, in addition to the mentioned characteristics, is also heat treatable and weldable, making it useful for a wide range of industrial applications [8]. Metallurgical bonding is obtained by diffusion processes between parts with mutual interface. The diffusion process is governed by temperature, pressure, surface quality, and bonding time [9].

One of the methods that results in metallurgical bonding is joining by plastic deformation, or “cold welding”, in which similar and dissimilar metals can be combined without melting. This method enables bonding large surfaces together, as opposed to small bonded areas obtained in mechanical bonding methods, such as riveting and clinching. Joining by plastic deformation also enables joining a wide range of materials (dissimilar included), with less distortion and residual stresses, and with high process reliability. A comprehensive review on the advantages and limitations of various plastic joining methods is given in [10]. 

Commonly used joining processes by plastic deformation include cold and hot roll-bonding [4,5,6,11,12,13], co-extrusion [14,15], and compression [16,17,18]. The effectiveness of such multi-layered composites as load-bearing components highly depends on the interface strength between the different metallic layers [19,20]. There are various methods proposed in the literature for the quantification of bonding strength. In [5], the bond strength of hot-rolled Al–Mg–Al composites was investigated. The hot rolling was conducted at several pre-heated temperatures of 400–475°, with a single reduction pass of 60%, 70% or 80%. The average bond strength was estimated using dog-bone specimens cut in the rolling direction, with a maximum bond strength of 66 MPa reported for the lowest temperature and reduction utilized. In [4], the strength obtained in cold-rolled Al–St–Al strips was investigated. Thickness reductions of 10–65% were conducted, and the average bond strength was estimated using a peel test. It was reported that the increased reduction ratio resulted in a higher contact pressure and increased bond strength. The study in [4] reports two interesting findings. It was shown that increasing the roll speed, although it did not increase the contact pressure, resulted in an increase in the average bond strength. It was also reported that increasing the yield strength of the inner layer (using higher strength steel), although increasing the contact pressure, resulted in a lower bond strength. These findings may hint that interface deformation, rather than interface pressure, plays a critical role in bond formation. In a recent study, hot compression bonding of 2196 Al–Cu–Li alloys, at temperatures of 450–550 °C and an axial compression of 20–60% at different strain rates, was investigated [18]. Tensile specimens were machined from the compressed samples and tested, to estimate the average bond strength. The study defined two parameters to quantify the bond strength with respect to the original material strength and elongation. These are, in essence, a normalized maximum stress (max bond stress/max material stress) and normalized elongation (max bond elongation/max material elongation). It was shown that a scalar parameter, which is a function of temperature, strain, and stress values, can be correlated to the normalized bond strength. A similar approach was applied by the authors of this manuscript to define the bond criterion in hot-rolled Al–Al strips [21].

The main limitation of the various studies reported in the literature is the assumption that all of the interface is metallurgically bonded; this is not necessarily the case, as the thermo-mechanical fields that govern the formation of the metallurgical bond are usually inhomogeneous across the contact interface area [21]. This implies that the measured bond strength, which is computed as the peak load at debonding divided by the contact area, is only a crude estimation. For the design of optimal bonding processes, it is first necessary to determine how local thermo-mechanical field values, rather than global average values, influence the bond strength.

The goal of the current study is to correlate between the local thermo-mechanical fields that develop during hot compression bonding and the resulting metallurgically bonded areas. Tensile debonding experiments were conducted in order to expose the bonded interface, and characterize and quantify the areas that underwent metallurgical bonding. This analysis, in conjunction with finite element analysis, enabled correlation of the exposed bonded area to the thermo-mechanical fields, which develop during the bonding process.

The article is structured as follows: Section 2 describes the bonding and debonding experiments, metallurgical characterization, and finite element modeling. The experimental and finite element analysis results are described in Section 3. Section 4 is devoted to discussing the findings, and correlating between the thermo-mechanical fields and the exposed surface areas. The summary and conclusions, as well as the plans for future expansion of the study, are given in Section 5.

## 2. Materials and Methods

To investigate the relation between the bonding surface and the thermo-mechanical fields that govern the bonding process, an experimental/computational methodology was utilized. First, compression experiments at different test temperatures were conducted in order to obtain metallurgical bonding between the specimens. To quantify the thermo-mechanical fields, which develop at the bonding interface, finite element modeling of the bonding tests was performed, verified and validated. Next, debonding experiments were conducted in order to both asses the interface bonding strength and to expose the bonded surfaces for metallurgical characterization. 

### 2.1. Bonding and Debonding Experimental Setup and Metallurgical Charcterization

**Figure 1:**
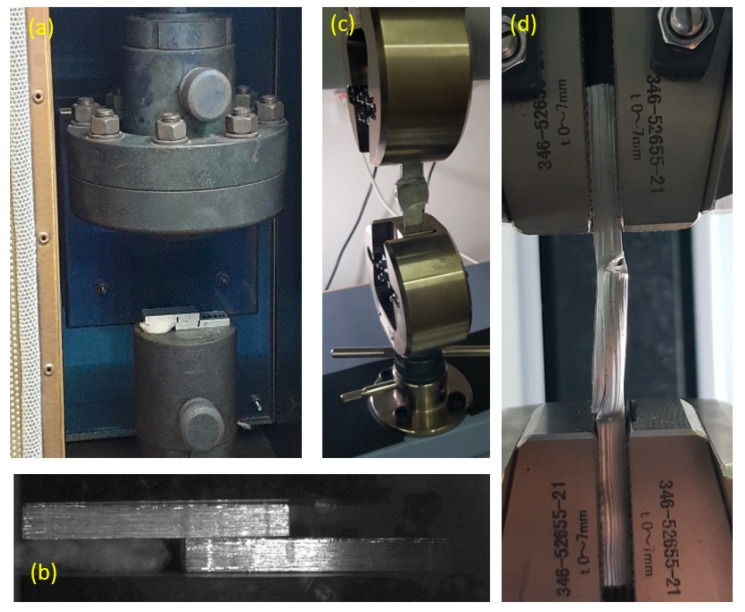
A specimen in the compression experimental system (**a**), and initial beam configuration in the compression tests, placed within the system (**b**). A specimen in the tensile machine (**c**), also shown from a side view inside the tensile grips (**d**).

In the present study, beams of Al6061 were cut from a flat plate (cold rolled with surface quality N6) to approximate dimensions of 6.5 × 10 × 58 mm each. Hot compression tests were conducted in an MTS exceed system machine with maximal load of 300 KN, equipped with an environmental chamber of up to 550 °C. In order to create diffusion bonding using plastic forming, pairs of Al6061 beams were placed one on top of the other with overlap of 20 mm and compressed to different degrees of axial deformations under 300, 400 or 500 °C using a constant ram velocity of 1 mm/min (see Figure 1a). The non-overlapping part of the beams was intentionally left in order to enable consecutive tension tests for examining the debonding between the beams. The initial configuration of the beams in the compression tests is presented in Figure 1b. Special attention was given to collinear placement of the beams, one relative to the other. The specimen deformation during the bonding and debonding process was monitored using a high-resolution Manta G-319 camera. The time-dependent deformation patterns were subsequently used to validate the computational models as will be discussed later on.

In order to ensure that the system tools and the specimen had reached the target temperature (so that the experiment could be considered approximately isothermal), the furnace was held at the target temperature for 40 min. Then the two beams were placed on the lower pressing plate (with no pressure applied), and held for additional 20 min before beginning the compression. 

The debonding tensile tests were conducted using a 10 kN Shimadzu electro-mechanical test machine. All tension experiments were conducted at room temperature (and after the specimens were completely cooled) with a loading rate of 1 mm/min, and all tests were conducted until failure. Following the debonding tests, a scanning electron microscope (SEM, JEOL JSM-7400F, Tokyo, Japan) was used to examine the exposed fracture surfaces and assess the different bonding zones.

### 2.2. Computational Modeling

**Figure 2:**
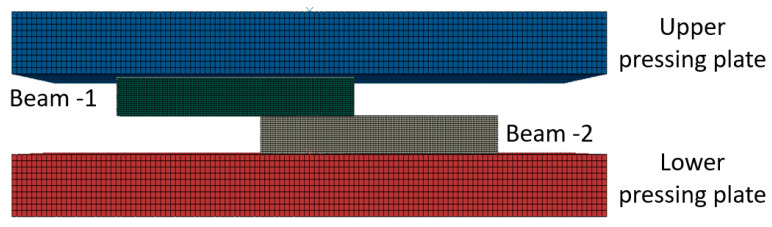
The geometry of the compression system represented in the model, with the 3D mesh used throughout this study (side view).

As described in Section 2.1, the compression tests were performed within a furnace, which is a part of the MTS system. Special attention was given to ensure both system tools and the specimens had reached the target temperature. Since the experiments were conducted under low ram velocities, the heat generation due to plastic dissipation can be neglected and it may be assumed that the bonding process is isothermal. The computational models were developed using the commercial FE (finite elements) code ABAQUS using an explicit solver. The Al6061 was defined as an elasto-plastic material using the J_2_ yielding criterion with isotropic hardening. The flow curves were characterized using compression tests on single-beam specimens, at all temperatures relevant to this study (25, 300, 400, and 500 °C). Since digital image data were used to track the displacement of the surfaces of the pressing plates in contact with the specimen, the elastic response of the tools became irrelevant and the pressing plates were defined in the FE model as rigid. A penalty-type contact constraint was specified between the beams (at the interface) and also between the beams and pressing plates. In the tangential direction a constant friction coefficient of 0.4 (at all temperatures) was defined. The friction coefficient between Al and steel was previously characterized by the authors in [22]. No penetration was allowed in the normal direction (“hard contact” definition). Due to symmetry, only half of the system was modeled as shown in Figure 2. The mesh was constructed using 8-nodal hexahedral elements, with reduced integration.

The experimentally measured displacement was prescribed as the boundary conditions on the upper pressing plate while the lower pressing plate was clamped. The computational models underwent standard convergence tests to verify the solution was converged with respect to element size and time step (see Appendix A). 

## 3. Results

### 3.1. Determination of Al6061 Flow Stress

As was mentioned in Section 2.1, the system tools and the specimen were held at the target temperature for 40 and 20 min, respectively, before starting the compression, so that the experiment could be considered isothermal. However, since a wide range of temperatures is considered in this work, characterization of the AA6061 flow stress at these temperatures is necessary for the computational study.

The flow stress of the material was characterized using compression tests of single-beam specimens, at 25, 300, 400, and 500 °C. An FE model was built for each of the test temperatures, and the flow stress was determined using the iterative approach detailed in [22]. The resulting stress–strain relations are provided in Table 1, in the following form: $\sigma =K\cdot \varepsilon^{n}+\sigma_{yield}$.

It can be seen from Table 1 that the flow stress decreases as the temperature increases, as expected. The strain hardening (represented by the slope of the stress–strain curve) is significant at room temperature and small at elevated temperatures. The strain hardening is also monotonically decreasing with increasing temperature. It should be noted that the constant representing the yield stress at different temperatures is similar to the values of the yield stress reported in the literature for Al6061-T6 and Al6061-T651 [23].

### 3.2. Compression Bonding Tests

The experimental program is presented in Table 2, along with the axial reductions obtained in the compression bonding stage.

**Figure 3:**
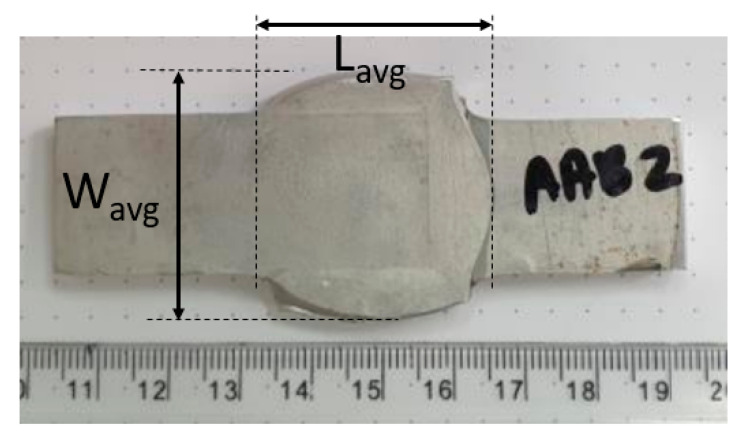
Representative measurements of the overlapping area.

It can be seen from Table 2 that, at the smallest reductions, bonding was not achieved at 500 °C; however, it was achieved at 400 °C and 300 °C. The dimensions of the overlap area are indicated in Figure 3, using an example of specimen AAA2 after the compression.

**Figure 4:**
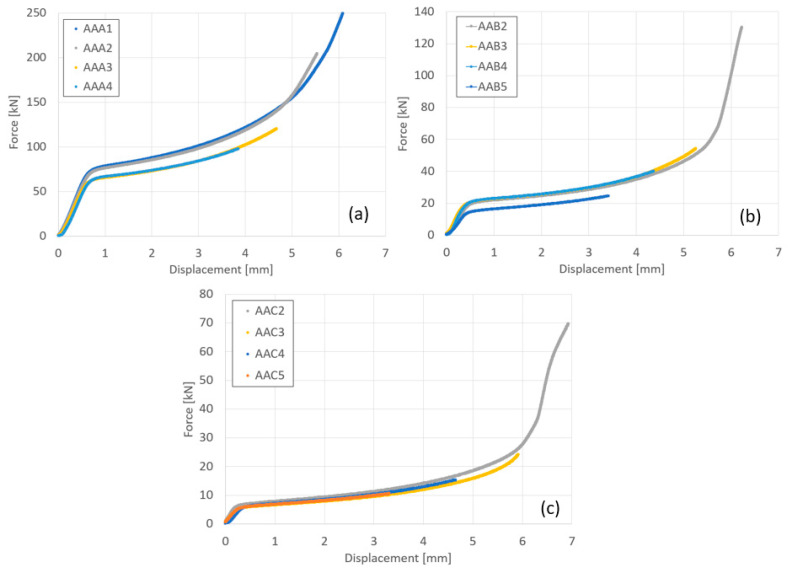
Force–displacement curves of the couples of Al6061 compressed at the following different temperatures: 300 °C (**a**), 400 °C (**b**), and 500 °C (**c**).

The force–displacement curves obtained in the compression bonding tests, at 300, 400 and 500 °C, are presented in Figure 4 for the entire range of reductions examined. It can be seen that the curves obtained for the specimens that were compressed at the same temperature conditions are overlapping, as expected. Also, as the temperature increases the forces required to obtain the same amount of deformation decrease.

It should be noted that the differences in the force–displacement curves of the specimens AAA3,AAA4 compared to AAA1,AAA2 result from differences in the length of the overlap (see Figure 1) when positioning of the specimens in the furnace for the compression test (which was significantly smaller for specimens AAA3,AAA4).

### 3.3. Validation of the Finite Element Models

**Figure 5:**
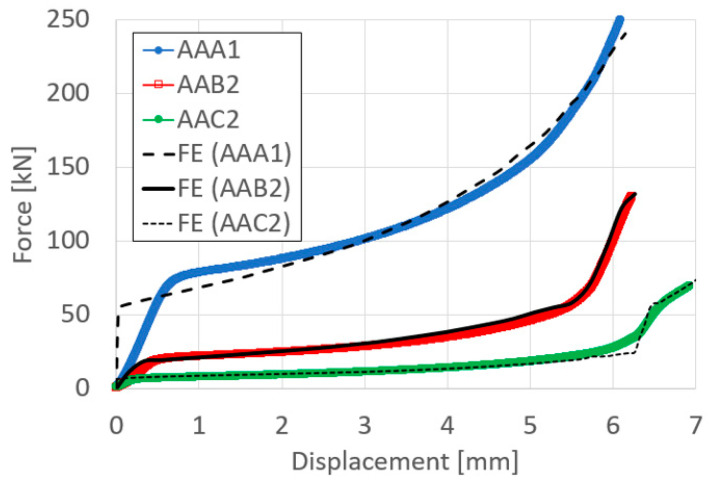
Experimental Vs computed force–displacement curves of the couples of Al6061 compressed at the following temperatures: 300 °C, 400 °C, and 500 °C.

To validate the computational models, a comparison between the measured and computed values was performed. As an example, the results for the largest reduction at each tested temperature are presented in Figure 5.

**Figure 6:**
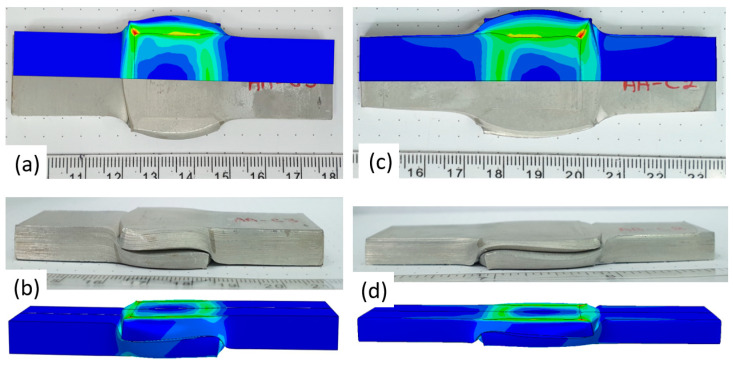
Upper (**a**) and side (**b**) view of specimen AAC3. Upper (**c**) and side (**d**) view of specimen AAC2.

Figure 5 demonstrates that the computational model is able to follow the experimental force–displacement curves. In addition to the force–displacement curve, the computed deformed specimen dimensions were compared to the experimentally obtained deformation. Representative examples are presented in the following Figure 6, which show that the deformation contours fit the experimentally obtained ones.

### 3.4. Debonding Experiments

As previously mentioned, the debonding experiments were conducted mainly in order to expose the bonded surfaces. Nevertheless, the shear stress required for delamination (which is equal to the bonding strength under shear) was roughly approximated by $\tau_{bonding}\approx F_{\max}/A_{0}$, where $F_{\max}$ is the maximum force obtained in the tension test and $A_{0}$ is the approximated overlapping area obtained in the compression stage (see Table 2 for the width and the length of the interface area measurement after compression). The results of the approximated $\tau_{bonding}$ are presented in the following Table 3. It is important to notice that the approximated $\tau_{bonding}$ is computed under the assumption that the entire overlapping area $A_{0}$ is bonded with the same strength, which may be a rough approximation. Furthermore, only a fraction of the overlapping area may be bonded, as discussed in the following section, which microscopically examines the interface after failure.

### 3.5. Macroscopic Charcterization Following Debonding Tests

**Figure 7:**
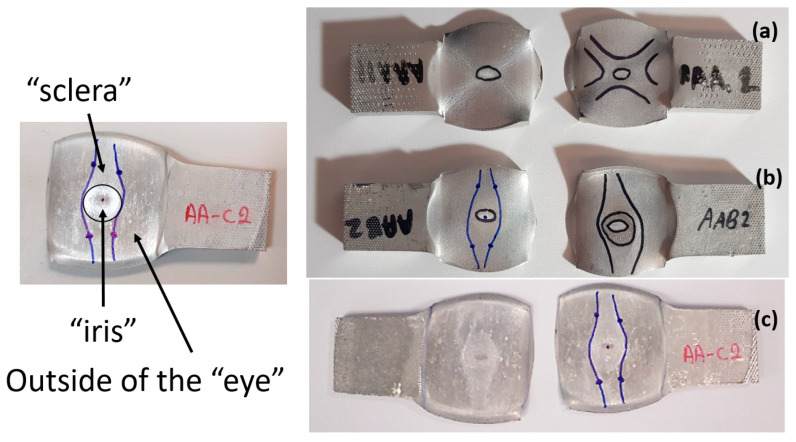
Examples of the interfaces revealed after debonding by a tension test. Specimen AAA1 (bonded at 300 °C, 46% reduction) (**a**) Specimens AAB2 (bonded at 400 °C, 49% reduction) (**b**), Specimen AAC2 (bonded at 500 °C, 53% reduction) (**c**).

Following the debonding of the beams by the tension test, the revealed interface has several distinguishable areas, which can be observed without any equipment. As can be seen from Figure 7, the same general pattern can be observed in the specimens that were bonded at different degrees of reduction (see Figure 7a–c), as well as at different temperatures. This macroscopic pattern resembled an eye, and included a rhombus-like area (“sclera”, outlined on the specimen surface in blue in Figure 7), which contains a round inner “iris” area (see Figure 7b). The macroscopic pattern is a bit different at 300 °C, where the region of the “sclera” resembles the letter “X” rather than a rhombus. In the specimens that were bonded at the same temperature, the macroscopic patterns are similar (however their dimensions change with different reductions). 

The division into different areas is thought to result from different surface textures at the interface, which differ in degrees of roughness compared with their surroundings, and therefore reflect light differently. The different areas were also examined microscopically, as discussed in the following subsection. 

### 3.6. Microscopic Charcterization Following Debonding

Based on the macroscopic observations presented in Figure 7, it was initially speculated that the interface eye-like pattern revealed by the debonding is a result of differences in textures, which have a microscopic as well as a macroscopic distinction (which causes the differences in light reflection).

The three areas—“iris”, “sclera”, and the outside of the “eye”—were examined using a SEM microscope, in specimens that were bonded at different temperatures (300 °C, 400 °C, 500 °C), and also ones that underwent different reductions at the same temperature. The different areas were magnified up to ×2500 in order to reveal differences in the textural characteristics at the microscopic level. Representative examples of each area are presented in the following Table 4. Those examples are of specimens that were bonded at different temperatures, but with a similar degree of reduction. Specimen AAA1 was bonded at 300 °C and 46.5% reduction, AAB2 was bonded at 400 °C and 49.5% reduction, and AAC3 was bonded at 500 °C and 45% reduction.

It can be seen from Table 4 that the microscopic features indicate a diffusional bond was created; however, not always along the entire interface. The presence of dimples, which are characteristic for ductile failure (of material bulk), are evidence for the diffusional bonding that took place during the compression stage. In general, deeper dimples will be correlated with a stronger diffusional bond. The interface microscopic features also depend on the debonding method, which was obtained by shear loading.

In the AAA1 sample, obtained at 300 °C, the “Iris” is covered with dimples of various degrees of depth, some of which appear to be quite deep and large, and others are shallower. Their round shape indicates that this area failed in tension. At the “sclera”, only a part of the area is covered by dimples. At smaller magnifications (which are not included in Table 4), it seems the stripes of dimples concentrate in the vicinity of grain boundaries. In between the stripes of dimples the areas seem to resemble a brittle-like surface, with ripples that imply the presence of a mechanical bonding as well (in which case both sides of the interface grip each other through the irregularities in the surface). Outside of the “eye”, the fracture surface is mostly brittle with visible grains (intergranular brittle fracture) and small patches of shallow dimples. Ripples indicating mechanical bonding are present throughout this area.

In the AAB2 sample, obtained at 400 °C, the “iris” is covered with very dense and deep dimples, indicating a strong diffusional bond. The dimples are slightly elongated, indicating that this area failed under a combination of tension and shear. The “sclera” is also mostly covered with dimples, which are very elongated as a result of the debonding process under shear. In between the dimpled area there are patches of tearing (cracking between primary voids), which create smooth surfaces. Outside of the “eye”, the fracture surface is partially covered with very shallow dimples, indicating a considerably weaker diffusional bonding. The rest of the surface is smoother, with outlines of intergranular surfaces, indicating an intergranular brittle fracture. Ripples can also be detected, indicating mechanical bonding took place as well.

In the AAC3 sample, obtained at 500 °C, the “iris” is covered with shallow dimples, indicating a weak diffusional bonding. Their round shape indicates that this area failed primarily in tension. The “sclera” looks similar to the same area in 400 °C, mostly covered with elongated dimples as a result of the shear debonding; however, the dimples are larger compared with the 400 °C “sclera”. Also present here are small areas of tear between the dimples. Outside of the “eye”, the fracture surface is covered with dimples, mostly shallow, but some areas have deeper dimples. In contrast with the lower temperatures, this indicates the entire area outside the “eye” was bonded by diffusion, though shallow dimples indicate a weak bonding strength. The round shape of the dimples indicates that this area failed mainly in tension. Underneath the dimples, ripples are also observed, indicating mechanical bonding took place as well.

The change in features obtained at similar reductions at different temperatures is assumed to result from the following two competing processes: the temperature-dependent diffusion on the one hand, and the temperature-dependent oxidation rate on the other, both of which increase with temperature. Before compression, the specimens are inserted into the furnace, and are held for an additional 20 min after arriving at the destination temperature. This allows time for the oxidation layer to build up. The oxidation rate in aluminum and its alloys is larger as the temperature increases [24]. In order to obtain diffusional bonding, the brittle oxidation layer needs to be broken in order to expose oxidation-free aluminum. The ram velocity is constant; however, since the strain distribution along the interface is non-uniform (see Section 4), the time required for achieving base metal-to-base metal contact will change at different areas along the interface. When such contact is achieved, the temperature is again an important factor for determining the diffusional bond strength, since the diffusion coefficient is exponentially dependent on temperature [9]. The local pressure also influences the diffusion coefficient (which increases with pressure), and by itself depends on the temperature, also through the flow stress. It seems, from the resulting interface in Table 4, that the strongest diffusion bonding was achieved at 400 °C. At 300 °C, the weaker bonding is probably due to the higher flow stress, which decreases the formability, whereas at 500 °C the weaker bonding is assumed to result from a thicker oxidation layer that has to be broken in order to enable base metal-to-base metal contact, which is required for the diffusion bonding to take place.

These results are conflicting with the approximated bonding strength in Table 3, which shows increasing strength with decreasing temperature. There are several causes for this inconsistency. One of them is that the simple calculation $\tau_{bonding}\approx F_{\max}/A_{0}$ assumes that the entire interface is bonded. We can see from Table 4 that this is not the case. Moreover, the microscopic features presented in Table 4 indicate that the bonding strength is not uniform along the bonded portions of the interface. In addition, the debonding process takes place at room temperature, after the specimens went through a heat treatment at different temperatures (300–500 °C, during pre-heating and compression). Since Al6061 is sensitive to heat treatments, the flow stress at room temperature will be different, which, in turn, also influences the resulting stress required for debonding. As mentioned in the introduction, there are several tests that are commonly used to determine the debonding force (such as peel or shear tests). Using a similar calculation, such as $F_{\max}/A_{0}$, on those tests overlooks the aforementioned considerations, thus leading to erroneous conclusions.

## 4. Correlation between Computed Thermo-Mechanical Fields and Microscopic Observations

In the previous section, the debonded interface was divided into three macroscopically distinguishable areas—the “iris”, “sclera”, and the outside of the “eye”. In order to obtain a better understanding of the thermo-mechanical fields that influenced these macroscopic features and created the different surface textures, FE models of the specimens, compressed at 300 °C, 400 °C and 500 °C to different degrees of reduction, were created. Mechanical fields, such as the principal components of the strains and stresses, were examined along the interface. Out of those, the ones that create contours that resemble the contours of the “eye” are presented in Table 5, for several specimens. It is important to mention that the FE thermo-mechanical models were only of the compression stage (i.e., the stage of bonding creation), and the mechanical fields presented in Table 5 show the fields iso-contours when reaching the final reduction in the specific specimen.

Table 5 shows the iso-contours of strain fields of several specimens at the end of the compression test. Those include components of the principal strains and shear strain. The interface between the beams at the beginning of the test lies on the x–z plane, so at the beginning of the test, the maximal principal (largest tension) ε11 component is εzz, the mid principal strain ε22 is εxx, and the minimum principal strain ε33 is the compression εyy.

It can be seen from Table 5 that the macroscopic features that create the contour of the “eye” seem to be a superposition of the influences of the three principal strain components—the approximately in-plane tension components ε11 and ε22, and the compression ε33 (normal to the interface). The relative magnitude between the components determines the dominant features on the surface, which are connected to the principal strain component iso-contours. The shape of the “eye” seems to be dominated by the ε22 component. The “iris” seems to be associated with both ε11 and ε33. The “×” macroscopic pattern at 300 °C seems to be a result of a dominant ε11 component (which reaches higher values at 300 °C).

The specimens AAC3 and AAC4 are both specimens that were bonded at 500 °C, but reached different reductions—45% (AAC3) and 32% (AAC4). Contrary to similar reductions at 400 °C and 300 °C, bonding was not created in specimen AAC4 (the two beams did not hold together when removed from the furnace). Accordingly, there was no detectable macroscopic pattern on the interface. In Table 5, the scale of the mechanical fields is similar, so that they could be compared. The values of ε11, ε22, and ε33 across the interface are clearly smaller in specimen AAC4 than in AAC3. It is assumed that there is a certain degree of strain that has to be locally reached in order for the oxidation layer to be broken. These strains lead to surface exposure and enable the base metal-to-base metal contact required to create a diffusion bond between the surfaces [25]. The deformation of specimen AAC4 was apparently insufficient for this degree of local strain to be reached. Since the thickness of the oxidation layer is temperature (and time) dependent, this local strain required for bonding is temperature dependent as well. This is consistent with the experimental results, which show that bonding was obtained at similar and even smaller reductions, in compression tests at lower temperatures, in which the oxidation layer is expected to be thinner.

**Figure 8:**
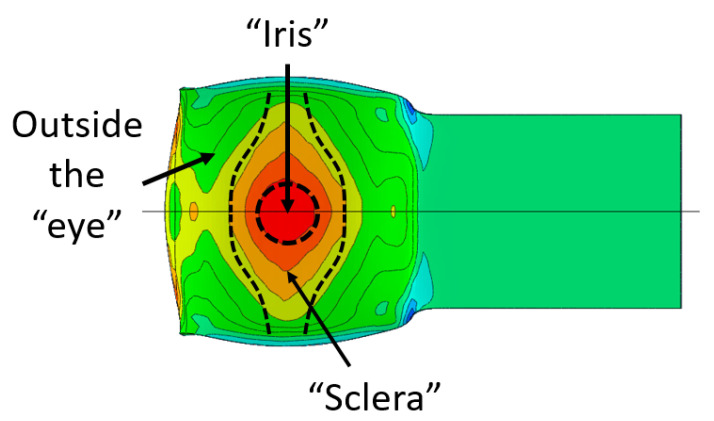
Areas of interest where the different values of bonding quality parameter *J* where computed.

**Figure 9:**
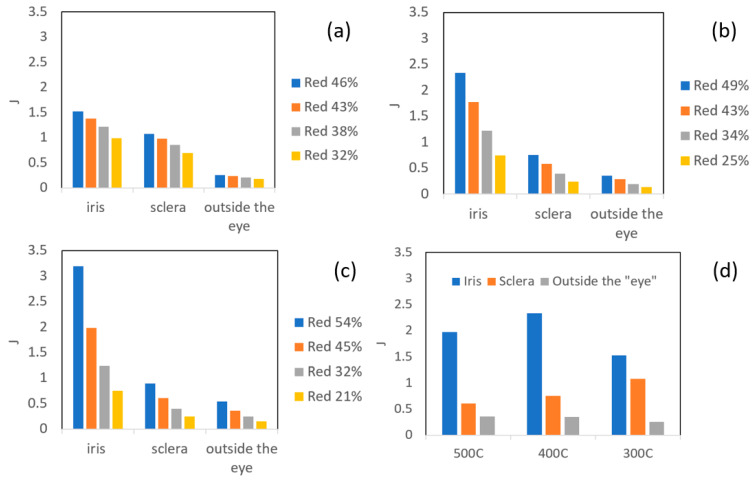
Comparison between bonding quality parameter *J* at the following temperatures: 300 °C (**a**), 400 °C (**b**), 500 °C (**c**) at different reduction values. *J* at different temperatures with similar reduction of about 45% (**d**).

To quantify the influence of the thermo-mechanical fields on the bonding conditions, the scalar bonding quality criterion used in [18] was utilized, as follows:

(1) $$J=\int_{0}^{t}k_{0}\frac{\sigma_{p}}{\sigma_{eq}}\exp\left(\frac{RT}{Q}\right)\dot{\varepsilon }\;dt$$

where $\sigma_{p},\sigma_{eq}$ are the pressure and equivalent stress, respectively, $\dot{\varepsilon }$ is the stain rate, *T* [K] is the temperature, *R* [J/mol K] is the universal gas constant and *Q* [J/mol] is the activation energy for diffusion. The value of $k_{0}$ depends on the material and surface conditions. The bonding quality is assumed to increase for greater values of *J*, with some critical value *J_cr_* denoting full metallurgical bonding. Unlike the study in [18], which utilized the average values from the experiment, in the current study, the local values of *J* were evaluated using the computed time-dependent mechanical fields values. Average values of *J* in each of the regions of interest, shown in Figure 8, were computed (*J* was calculated in differential form at five nodal points in the FE model, sampling each area). The results are provided in Figure 9, where $k_{0}$ was taken as 1 and *Q* was taken as 124–144 [J/mol] for 300–500 °C, respectively.

It can clearly be seen in Figure 9a–c that the computed average values of *J* are considerably higher in the “iris” region, for all temperatures considered. The *J* value decreases in the “sclera” and in the area outside of the “eye”. It can also be seen that the trend in *J* values between the different regions is maintained for all temperatures; however, the differences between the regions are greater as the temperature increases. It can be seen in Figure 9d that the average value of *J* in the “iris” is greater at 400 °C compared to the value at 500 °C. This correlates to the findings shown in Table 4, which show the highest density of dimples in the “iris” at 400 °C, compared to 500 °C and 300 °C. Figure 9d also shows that similar values of *J* outside the “eye” are obtained for 400 °C and 500 °C, with smaller values for 300 °C. These findings also correlate well with the metallurgical observation, which shows the presence of shallow dimples at 400 °C and 500 °C, and almost no evidence of metallurgical bonding at 300 °C. Nevertheless, the high value of *J* in the “sclera” at 300 °C, compared to 400 °C and 500 °C, does not seem to correlate with the metallurgical observations, since at 300 °C only a fraction of the area shows evidence of metallurgical bonding. Although the experimental/computational methodology presented in this study seems promising for identifying the relation between the interface conditions and the resulting bonding quality, more experimental data is required in order to determine the critical value of *J* for full metallurgical bonding in Al6061.

## 5. Summary and Conclusions

Experiments, in conjunction with finite element modeling and metallurgical characterization, were used to investigate the hot compression bonding of Al6061 beams. Temperatures of 300–500 °C and deformations of up to 60% were considered. Tensile debonding tests were conducted in order to expose which section of the interface was actually bonded, and to obtain what is commonly considered the bonding strength. SEM imaging was used to identify metallurgically bonded regions at the interface. It was revealed that the actual bonded area is much smaller than the interface contact area. This finding makes standard quantification of the bonding strength by $F_{\max}/A_{0}$ underestimate the true bonding strength. It was also shown that the bonded area follows a very distinct pattern, which is temperature dependent. The finite element modeling demonstrated that the principal plastic strain components, which develop across the interface, have similar patterns. The results from the finite element models were also used to compute a scalar bonding parameter *J*, which is deformation, temperature and time dependent. Good correlation was shown between the spatial distribution of the computed bonding parameter *J* and the microscopically observed inhomogeneity of the bonded areas across the interface. Future work will include modeling of the debonding experiments, using the cohesive zone approach to model the interface areas in order to provide a better understanding of the variations in bonding strength.

## Figures and Tables

**Table 1:** The flow stress relation characterized for the examined Al6061 at different temperatures.

**Specimen Temp. [°C]**	**25**	300	400	500
Flow stress [MPa]	$325\cdot \varepsilon^{0.75}+225$	$55\cdot \varepsilon^{0.8}+88$	$10\cdot \varepsilon^{0.75}+42$	$10\cdot \varepsilon^{0.8}+10$

**Table 2:** Compression bonding experimental parameters and dimensions of the specimens after the test.

Temperature [°C]	Specimen No.	Reduction [%]	W_avg_ [mm]	L_avg_ [mm]
300	AAA1	46.48	34.2	28.2
	AAA2	43.54	31.47	27.95
	AAA3	38.33	29.65	23.2
	AAA4	32.39	26.75	22.52
400	AAB2	49.45	34.5	26.50
	AAB3	43.39	31.50	25.56
	AAB4	34.29	28.98	23.70
	AAB5	25.02	25.60	21.50
500	AAC2	53.45	37.50	28.75
	AAC3	44.84	32.80	23.4
	AAC4	-	-	-
	AAC5	-	-	-

**Table 3:** Approximated $\tau_{bonding}$ from force obtained in tension and area from the compression stage.

Temperature [°C]	Specimen No.	Actual Reduction [%]	$\tau_{bonding}\;[\mathrm{MPa}]$	Notes
300	AAA1	46.48	12.16	
	AAA2	43.54	11.19	
	AAA3	38.33	5.08	
	AAA4	32.39	4.26	
400	AAB2	49.45	10.89	
	AAB3	43.39	11.73	
	AAB4	34.29	8.28	
	AAB5	25.02	7.22	
500	AAC2	53.45	7.29	
	AAC3	44.84	7.50	
	AAC4	-	-	No bonding
	AAC5	-	-	No bonding

**Table 4:** SEM images of different areas of the interface after debonding, magnification ×2500.

	“iris”	“sclera”	Outside the “eye”
AAA1 (Bonded at 300 °C, 46.5% reduction)	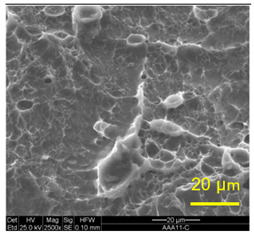	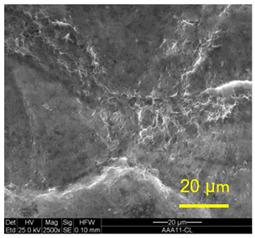	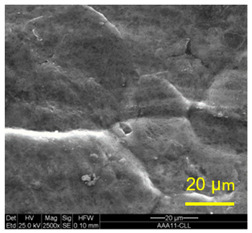
AAB2 (Bonded at 400 °C, 49.5% reduction)	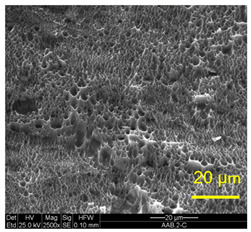	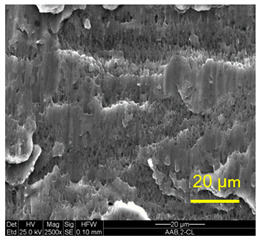	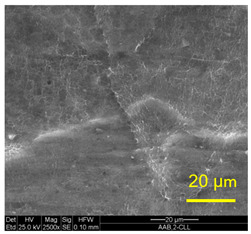
AAC3 (Bonded at 500 °C, 45% reduction)	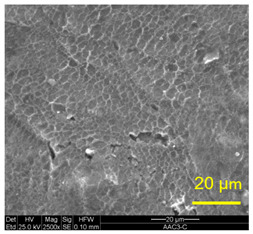	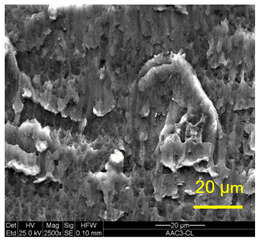	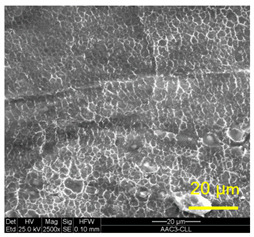

**Table 5:** Mechanical fields from FE models of the bonding interface when reaching the specimen’s final reduction.

Spec.	AAA1 (Bonded at 300 °C, 46.5% Reduction)	AAB2 (Bonded at 400 °C, 49.5% Reduction)
Macro image	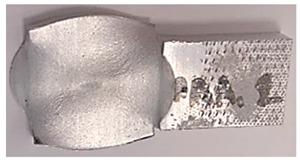	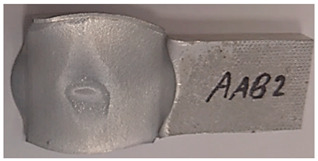
ɛ11 (Max. principal strain)	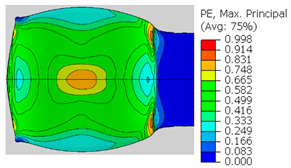	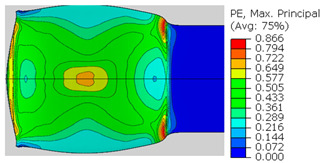
ɛ22 (Mid principal strain)	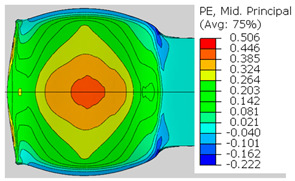	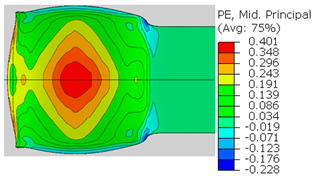
ɛ33 (Min. principal strain)	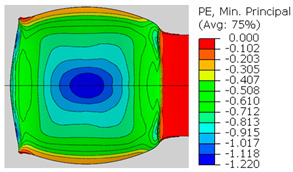	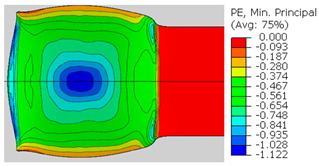
Spec.	AAC3 (Bonded at 500 °C, 45% reduction)	AAC4 (Bonded at 500 °C, 32% reduction)
Macro image	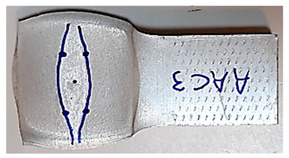	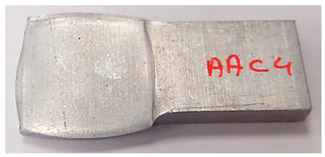
ɛ11 (Max. principal strain)	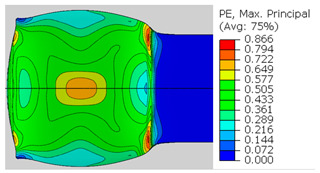	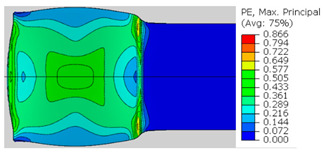
ɛ22 (Mid principal strain)	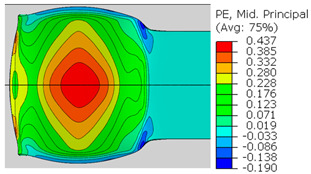	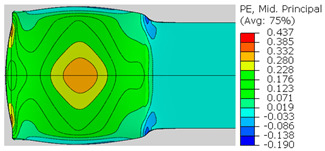
ɛ33 (Min. principal strain)	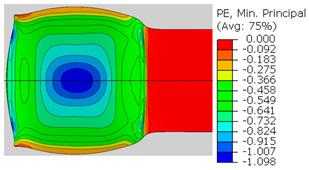	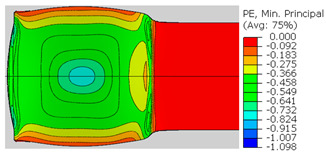

**Figure A1:**
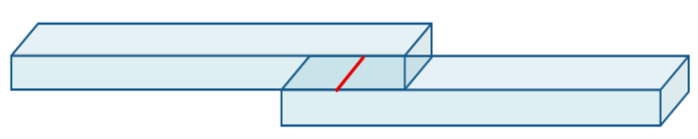
The two bars in contact before deformation. The red line indicates the path at the center of the contact area, along which the mechanical fields were extracted.

**Figure A2:**
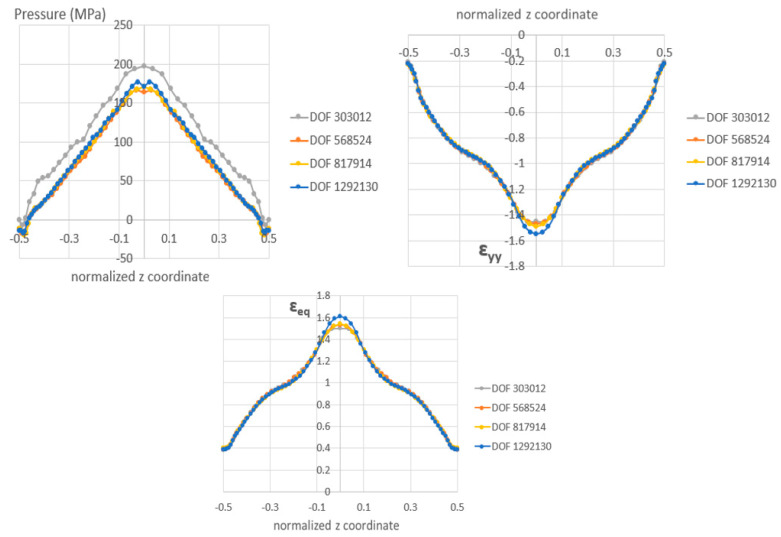
An example of a convergence test for the case of compression at 400 °C to 56% reduction. Here, for the chosen mass scaling value of 100,000, the mechanical fields (pressure—top, εyy—middle, and εeq—bottom) are examined for different degrees of mesh refinement.

## Data Availability

The data presented in this study are available on request from the corresponding author.

## Appendix A. Verification of the Computational Models

To ensure that the discretization error is minimal, convergence tests were performed. An explicit solver was used with mass scaling, so the convergence was checked for both the mesh refinement (increasing number of degrees of freedom—DOFs) and for different values of mass scaling for the chosen mesh. The two compressed bars were uniformly meshed so that the elements had an aspect ratio of one, which was kept for all four degrees of mesh refinement. As a test case, convergence was checked at 400 °C after a 56% reduction in the bars (the largest reduction obtained in the tests). The pressure, the strain component at y direction (εyy), and the equivalent plastic strain (εeq) (all influencing the bonding strength) were extracted along the width, at the center of the contact area (see Figure A1), at the end of the compression (at maximum displacement). In addition, the force–displacement response was also examined. For the chosen value of mass scaling (100,000), it was ensured that throughout the compression process the kinetic energy was at least 100 times smaller compared with the internal energy. The mesh included about 3 × 10^5^–1.3 × 10^6^ DOFs, respectively, for the entire model. The mesh used throughout this work included about 5.7 × 10^5^ DOFs, and a mass scaling value of 100,000. Figure A2 demonstrates that convergence was achieved for the chosen mesh. A similar examination was performed to ensure that the chosen mass scaling value also provides converged results.
